# The ABCG2 Transporter Affects Plasma Levels, Tissue Distribution and Milk Secretion of Lumichrome, a Natural Derivative of Riboflavin

**DOI:** 10.3390/ijms25189884

**Published:** 2024-09-13

**Authors:** Alicia Millán-García, Laura Álvarez-Fernández, Esther Blanco-Paniagua, Ana I. Álvarez, Gracia Merino

**Affiliations:** Department of Biomedical Sciences–Physiology, Faculty of Veterinary Medicine, Animal Health Institute (INDEGSAL), Campus de Vegazana, Universidad de León, 24071 León, Spain; amilg@unileon.es (A.M.-G.); lalvf@unileon.es (L.Á.-F.); eblap@unileon.es (E.B.-P.); aialvf@unileon.es (A.I.Á.)

**Keywords:** lumichrome, flavor, ABCG2, plasma levels, tissue distribution, milk secretion

## Abstract

The ABCG2 membrane transporter affects bioavailability and milk secretion of xenobiotics and natural compounds, including vitamins such as riboflavin. We aimed to characterize the in vitro and in vivo interaction of ABCG2 with lumichrome, the main photodegradation product of riboflavin, which has proven in vitro anti-cancer activity and a therapeutical role in antibacterial photodynamic therapy as an efficient photosensitizer. Using MDCK-II polarized cells overexpressing murine Abcg2 and human ABCG2 we found that lumichrome was efficiently transported by both variants. After lumichrome administration to wild-type and Abcg2^-/-^ mice, plasma AUC_20–120 min_ was 1.8-fold higher in Abcg2^-/-^ mice compared with wild-type mice. The liver and testis from Abcg2^-/-^ mice showed significantly higher lumichrome levels compared with wild-type, whereas lumichrome accumulation in small intestine content of wild-type mice was 2.7-fold higher than in Abcg2^-/-^ counterparts. Finally, a 4.1-fold-higher lumichrome accumulation in milk of wild-type versus Abcg2^-/-^ mice was found. Globally, our results show that ABCG2 plays a crucial role in plasma levels, tissue distribution and milk secretion of lumichrome potentially conditioning its biological activity.

## 1. Introduction

One of the key mechanisms that affects biodistribution and milk secretion of natural compounds and xenobiotics is the ATP-binding cassette transporter G2 (ABCG2), also known as breast cancer resistance protein (BCRP). It is expressed in the apical membrane of cells of organs that play a relevant role in biodistribution of compounds, such as the liver, kidney and gastrointestinal tract; the blood–brain, blood–placental and blood–testis barriers; and the lactating mammary gland [1,2]. It behaves as an efflux pump, playing a xenobiotic protective role in individual cells, in some organs and in the body as a whole [1,3]. Moreover, it modulates the absorption, distribution and elimination of drugs and natural and endogenous compounds [4,5], conditioning its pharmacokinetics and tissue distribution, lessening plasma levels of its substrates [1]. It is worth noting that it is the only ABC transporter that participates in the secretion of its substrates into milk [6].

Lumichrome, the main product of the photodegradation of riboflavin (vitamin B_2_ or lactoflavin) [7,8,9,10], is a natural compound present in the human body [11,12,13]. Coming from several sources, it is synthesized from riboflavin in the liver, kidney and digest-free small intestine [14] and produced by intestinal bacteria as a byproduct of the metabolism of riboflavin [15]. Out of the body, it can be efficiently produced by different microorganism strains such as *Microbacterium* sp. [16], *Pseudomonas riboflavina* [17], marine sponge-associated fungus *Acremonium persicinum* [18] and a species of *Norcardia* [19]. It can also be chemically synthesized [20,21] or synthesized by photodegradation of riboflavin in neutral or acidic conditions [8,11,22,23].

Regarding its biological effects, it is a nontoxic molecule [13,24] which acts as a competitive flavin reductase inhibitor [25,26]. Although the inhibition of this enzyme may interfere in the in vitro reduction of methaemoglobin to haemoglobin, it is believed that under normal conditions the enzyme scarcely contributes to that process [27].

It also inhibits, in a concentration-dependent manner, the riboflavin uptake by riboflavin carriers in HepG2 cells [28]. Additionally, it plays a therapeutical role in antibacterial photodynamic therapy as an efficient photosensitizer [11,29].

Lumichrome has proven anti-cancer activity in non-small-cell lung cancer (NSCLC) cell lines by suppressing growth and inducing apoptosis through p53-dependent mechanisms at concentrations above 25 µM [30]. It also shows significant cytotoxic activity against liver, breast and colorectal cancer cell lines (HepG2, MCF-7 and Colo-205, respectively), at concentrations higher than 40 µM [31].

In the presence of light, lumichrome efficiently produces singlet oxygen species [9], which induce the oxidation of sulfur-containing proteins and polyunsaturated fatty acids, [32,33] producing undesirable impairment of the sensorial properties of milk [34]. This may reduce milk nutritional value, making it unacceptable to consumers [35], since the sensorial characteristics of milk products are directly related to milk quality [36].

Riboflavin, the lumichrome precursor, is an in vitro and in vivo substrate of murine Abcg2 [37]. However, in vitro and in vivo interactions of ABCG2 with lumichrome remain unknown. Consequently, the aim of this study was to evaluate whether lumichrome is an in vitro substrate of ABCG2 and to describe the role of ABCG2 in plasma levels, tissue distribution and milk secretion of the aforementioned compound.

## 2. Results

### 2.1. Murine Abcg2 and Human ABCG2 Efficiently In Vitro Transport Lumichrome

**Figure 1:**
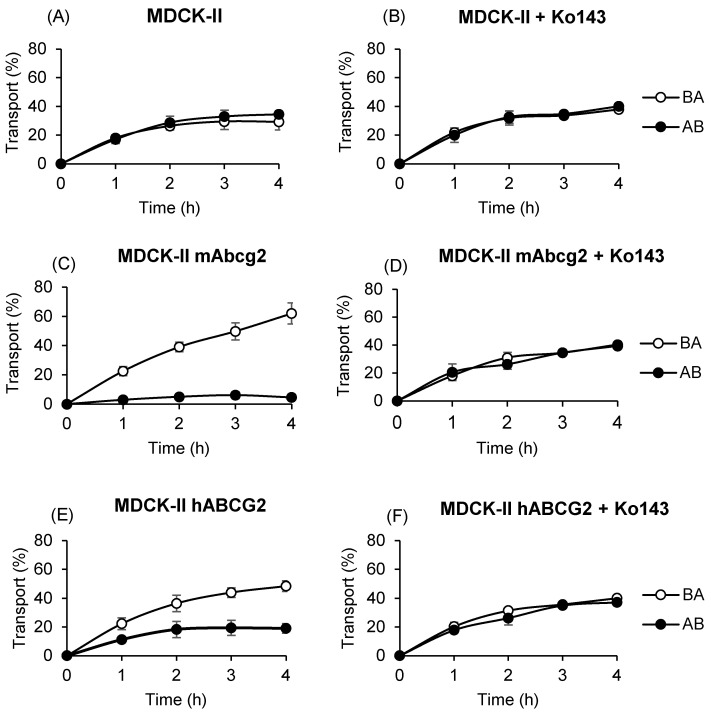
Transepithelial transport assay of lumichrome (10 µM) with or without the ABCG2-specific inhibitor Ko143 (1 µM) in parental MDCK-II cells (**A**,**B**) and their subclones transduced with murine (**C**,**D**) and human (**E**,**F**) ABCG2 variants. The experiment began by replacing the medium of both compartments with fresh culture medium with or without inhibitor and containing 10 µM lumichrome. Aliquots were taken at 1, 2, 3 and 4 h on the opposite side to where lumichrome had been added. All samples were stored at −20 °C until being analyzed by HPLC. Lumichrome appearance in the opposite compartment was presented as the fraction of the total compound added at the beginning of the experiment and expressed as a percentage. Results are shown as mean ± S.D.

To assess the role of ABCG2 in the in vitro transport of lumichrome, the MDCK-II parental cell line and its subclones transduced with murine Abcg2 and human ABCG2 were used, and vectorial transport of lumichrome across the monolayer was determined and relative efflux ratios were calculated (Figure 1, Table 1). The MDCK-II parental cell line displayed a similar transport pattern in either direction, apical to basolateral (AB) and basolateral to apical (BA) (Figure 1A,C,E), which is reflected in the relative efflux transport ratio (BA/AB) at 4 h (0.85 ± 0.13). However, murine Abcg2 and human ABCG2 subclones presented not only a higher basolateral to apical transport but also a lower apical to basolateral transport compared with parental cells, which resulted in significantly higher relative efflux transport ratios of 13.81 ± 3.25 (*p* < 0.001) and 2.57 ± 0.25 (*p* < 0.001), respectively.

The specificity of vectorial transport was checked using a specific inhibitor of ABCG2, Ko143. ABCG2-mediated transport was completely inhibited in all subclones (Figure 1B,D,F) which was reflected in similar relative efflux transport ratios between parental and transduced cells. These results show that lumichrome is efficiently transported by murine and human variants of the ABCG2 transporter.

### 2.2. Plasma Levels and Tissue Distribution of Lumichrome in Wild-Type and Abcg2^-/-^ Male Mice

To also evaluate the extent to which in vitro transport of lumichrome mediated by ABCG2 is reflected in vivo, plasma and tissue levels of lumichrome were measured in wild-type and Abcg2^-/-^ male mice after its intraperitoneal administration at a dose of 10 mg/kg. This setting was selected rooted in previous studies, where sham-operated or ovariectomized female mice were intraperitoneally injected with lumichrome [38]. We chose a dose of 10 mg/kg, since this is the minimum dose needed to detect the compound in biological samples by the chromatographic method.

**Figure 2:**
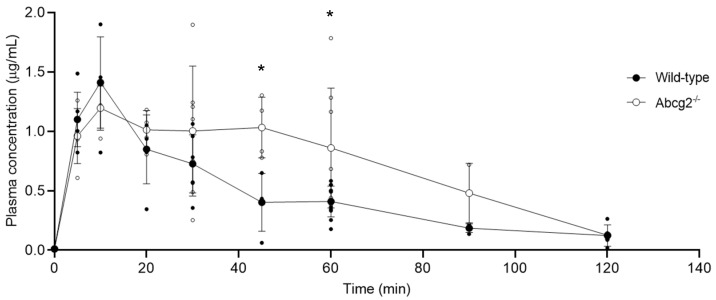
Plasma concentration after intraperitoneal administration of lumichrome (10 mg/kg) to wild-type and Abcg2^-/-^ male mice (*n* = 3−10). Plasma samples were collected at 5, 10, 20, 30, 45, 60, 90 and 120 min. Concentration of lumichrome was determined by HPLC analysis. Results are presented as individual data and mean ± S.D. (*) *p* ≤ 0.05: significant differences between both groups of mice.

The area under the plasma-concentration–time curve (AUC) showed significant differences between Abcg2^-/-^ and wild-type mice from 20 min to 120 min post-administration (Figure 2), being, in Abcg2^-/-^ mice, almost 2-fold higher than in the wild-type counterparts (1.29 ± 0.11 µG·h/mL vs. 0.72 ± 0.05 µg·h/mL; *p* = 0.045). Statistically significant differences were also found at the sampling time points of 45 min (0.39 ± 0.24 µg/mL in wild-type mice vs. 1.02 ± 0.26 µg/mL in Abcg2^-/-^ mice; *p* = 0.01) and 60 min post-administration (0.40 ± 0.13 µg/mL in wild-type mice vs. 0.85 ± 0.50 µg/mL in Abcg2^-/-^ mice; *p* = 0.02).

**Figure 3:**
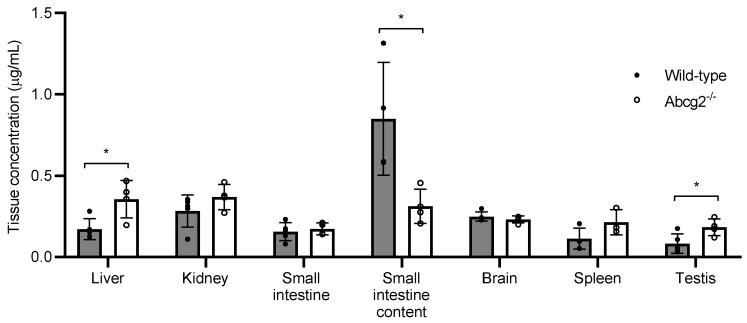
Tissue concentration of lumichrome in wild-type and Abcg2^-/-^ male mice 1 h after intraperitoneal administration of lumichrome at 10 mg/kg (*n* = 4−5). Results are presented as individual data and mean ± S.D. (*) *p* ≤ 0.05: significant differences between both groups of mice.

In order to further study the role of ABCG2 in the biodistribution and elimination of lumichrome, concentrations of lumichrome were analyzed in the following tissues: liver, kidney, small intestine, small intestine content, spleen, brain and testis 60 min after intraperitoneal administration at a dose of 10 mg/kg (Figure 3).

Lumichrome was widely distributed in all organs analyzed. Although at 60 min post- administration, plasma levels were 2.1-fold higher in Abcg2^-/-^ mice compared with their wild-type counterparts (Figure 2), lumichrome concentrations in small intestine content retrieved from wild-type mice were 2.7-fold higher than in Abcg2^-/-^ mice (0.85 ± 0.35 µg/mL vs. 0.31 ± 0.11 µg/mL). These results show that Abcg2 mediates the excretion of lumichrome into the intestinal lumen, contributing to its elimination.

Additionally, statistically significant differences in concentrations between both types of animals were found in the liver and testis (*p* = 0.02 and *p* = 0.03, respectively) (Figure 3). The liver and testis of Abcg2^-/-^ mice showed a 2.3-fold higher accumulation of lumichrome compared with wild-type mice (0.34 ± 0.12 µg/mL vs. 0.15 ± 0.07 µg/mL and 0.18 ± 0.05 µg/mL vs. 0.08 ± 0.06 µg/mL, respectively).

These results demonstrate that lumichrome is an in vivo substrate of the ABCG2 transporter and it affects plasma concentrations and tissue distribution of lumichrome.

### 2.3. Secretion of Lumichrome into Milk in Wild-Type and Abcg2^-/-^ Lactating Female Mice

To evaluate whether Abcg2 is involved in the secretion of lumichrome into milk, 10 mg/kg of this compound was administered intraperitoneally to lactating wild-type and Abcg2^-/-^ lactating female mice. Blood and milk samples were collected 30 min after administration.

**Figure 4:**
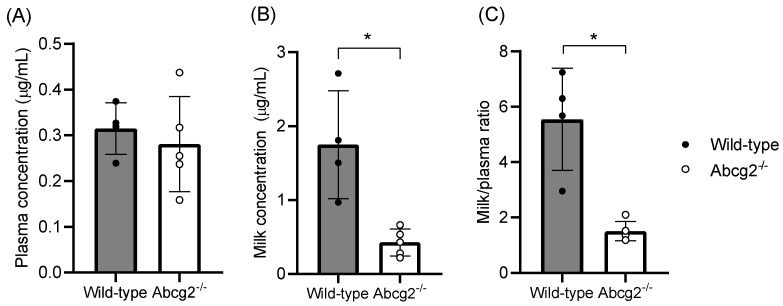
Plasma and milk concentrations and milk-to-plasma ratios of lumichrome in wild-type and Abcg2^-/-^ female lactating mice after intraperitoneal administration of lumichrome at a dose of 10 mg/kg body weight (*n* = 4−5). Plasma and milk samples were collected 30 min after intraperitoneal administration and concentrations were determined by HPLC. Results are presented as individual data and mean ± S.D. (*) *p* ≤ 0.05: significant differences between both groups of mice.

Similar plasma levels (Figure 4A) were found in wild-type and Abcg2^-/-^ mice (0.31 ± 0.06 µg/mL vs. 0.28 ± 0.10 µg/mL, respectively). Conversely, accumulation of lumichrome in milk from wild-type mice (Figure 4B) was 4.1-fold higher in comparison with Abcg2^-/-^ mice (1.75 ± 0.73 µg/mL vs. 0.43 ± 0.18 µg/mL; *p* < 0.01). In addition, the milk-to-plasma ratio of lumichrome (Figure 4C) in wild-type mice was almost 3.7-fold higher than in Abcg2^-/-^ mice (5.54 ± 1.84 µg/mL vs. 1.51 ± 0.35 µg/mL; *p* < 0.01). These results reveal that Abcg2 plays an important role in the active secretion of lumichrome into milk, absolutely confirming its in vivo interaction with the transporter.

## 3. Discussion

Lumichrome is an active derivative of riboflavin with relevant cytotoxic and prooxidant activities. Owing to the relationship between biological action and bioavailability, it is crucial to study the pharmacokinetics and systemic exposure of lumichrome and whether it is influenced by the presence of the efflux transporter ABCG2.

Accordingly, this study shows, for the first time, the in vitro interaction of lumichrome with the murine Abcg2 and human ABCG2 and how murine Abcg2 affects plasma levels, tissue accumulation and milk secretion of lumichrome.

In vitro transepithelial transport assays showed that lumichrome is efficiently transported by murine and human variants of the ABCG2 transporter, as well as other endogenous compounds like riboflavin [37]. The differences observed between murine and human ABCG2 transduced subclones correlated with the ones obtained for other ABCG2 substrates such as meloxicam [39] and albendazole sulphoxide [40], which could be attributed to a lower affinity with the compound of the human variant or a lower expression of the transporter in the aforementioned subclone [41].

In order to assess whether the in vitro results could be extrapolated to the in vivo situation, the concentration of lumichrome was determined in plasma and several tissues from wild-type and Abcg2^-/-^ male mice after its intraperitoneal administration at a dose of 10 mg/kg. Although we cannot discount the possibility that the estimation of lumichrome concentration might be affected by the degradation of endogenous riboflavin, the higher plasma AUC_20–120 min_ in Abcg2^-/-^ mice than in wild-type mice supports the idea that Abcg2 contributes to lumichrome elimination (Figure 2), as was the case for riboflavin [37]. Therefore, any changes in Abcg2 activity may alter the rate of elimination of lumichrome. This could have an impact on the success of its therapeutic use, such as in the case of photodynamic therapy, since lumichrome may be one of the photosensitizers chosen for the treatment of skin cancer and other diseases [42], for which systemic levels are therapeutically relevant. Changes in ABCG2 expression or functionality may alter the bioavailability and pharmacokinetics of ABCG2 substrates [2,43], potentially affecting their effects. This could be the case for lumichrome. Alterations in ABCG2 activity may be due to known genetic single nucleotide polymorphisms (SNPs), such as Q141K, which leads to a dysfunction of the transporter and has a high incidence rate among the Asian population [44]. Additionally, some natural [45] and dietary compounds including soy isoflavones [46,47] or flaxseed [48], and the coadministration of several drugs currently used in the treatment of human pathologies [49,50,51] may also decrease the activity of the transporter.

Regarding tissue distribution, our results show that lumichrome was distributed in all organs analyzed (Figure 3). The higher concentration of lumichrome in small intestine content retrieved from wild-type mice than from Abcg2^-/-^ mice (0.85 ± 0.35 µg/mL vs. 0.31 ± 0.11 µg/mL) confirms that Abcg2 contributes to lumichrome elimination. These results indicate that differences in concentrations in the intestinal lumen may be due to changes in Abcg2 activity which may have consequences for the potential use of this compound in colorectal cancer [31]. For instance, in this case, a decreased concentration of lumichrome in the intestine, potentially due to intestinal ABCG2 inhibition by dietary compounds, may result in unsuccessful chemotherapy.

In relation to the liver and testis, Abcg2^-/-^ mice showed a 2.3-fold-higher lumichrome accumulation compared with their wild-type counterparts in both organs (0.34 ± 0.12 µg/mL vs. 0.15 ± 0.07 µg/mL and 0.18 ± 0.05 µg/mL vs. 0.08 ± 0.06 µg/mL, respectively), which may be a reflection of plasma differences. Even so, these divergences indicate that changes in the activity of the transporter can affect the levels of lumichrome in these organs and, therefore, its cytotoxic effect in a potential use of this compound against liver cancer cells [31].

To study milk secretion of lumichrome, in vivo assays with wild-type and Abcg2^-/-^ female mice were performed. After the intraperitoneal administration of lumichrome at a dose of 10 mg/kg, similar plasma levels were found in both groups of mice (0.31 ± 0.06 µg/mL vs. 0.28 ± 0.10 µg/mL, respectively). Notwithstanding, accumulation of lumichrome in milk (Figure 4B) and milk-to-plasma ratio (Figure 4C) from wild-type mice was higher in comparison with Abcg2^-/-^ mice (1.75 ± 0.73 µg/mL vs. 0.43 ± 0.18 µg/mL; *p* < 0.01 and 5.54 ± 1.84 µg/mL vs. 1.51 ± 0.35 µg/mL; *p* < 0.01, respectively).

Higher concentrations of Abcg2 substrates in milk of wild-type compared with Abcg2^-/-^ mice were also observed after the administration of other natural substrates such as riboflavin [37], biotin [37], bile acids [52] or melatonin and its metabolites [53]. It has also been shown that the endogenous concentration of riboflavin in milk of wild-type mice was 63-fold higher than in the Abcg2^-/-^ counterpart [37]. Comparing these results with the ones obtained for lumichrome administration, it can be stated that ABCG2 transports riboflavin more efficiently than lumichrome. These differences may be related to the fact that ABCG2 transports polar molecules more efficiently than non-polar ones [54]. In fact, riboflavin has a ribityl side chain which enwidens its topological polar surface area compared with lumichrome (155Å² vs. 84Å², respectively) [55,56].

Although the transport of lumichrome may be mediated by solute carrier (SLC) transporters [57,58,59], the main ABC efflux transporter upregulated during lactation in the mammary gland is ABCG2, which corroborates that, unequivocally, Abcg2 plays a major role in the milk secretion of lumichrome, confirming the fact that lumichrome is an ABCG2 substrate. Compensatory changes in the expression of genes coding for proteins involved in transport in the Abcg2^-/-^ mice that could affect lumichrome biodistribution cannot be completely excluded. However, indications for such disturbing effects in previous studies have never been shown [60,61,62].

Changes in ABCG2 expression alter the concentration of ABCG2 substrates secreted into milk [6,53], including lumichrome, as we now report. Since this molecule affects sensorial properties of milk [34], any alteration in the transporter can change levels of lumichrome in milk, therefore potentially affecting milk quality [36].

In conclusion, our results support an important interaction between lumichrome and the ABCG2 transporter. This study shows, for the first time, that lumichrome is an in vitro substrate of murine and human variants of ABCG2. Furthermore, in vivo assays showed that Abcg2 actively participates in bioavailability, tissue distribution and secretion of lumichrome into milk.

## 4. Materials and Methods

### 4.1. Chemicals

Lumichrome, mitoxantrone dihydrochloride and Lucifer Yellow were purchased from Sigma-Aldrich (St. Louis, MO, USA). Ko143 and albendazole-2-amino sulfone were acquired from Tocris (Bristol, UK) and LGC Standards (Teddington, Middlesex, UK), respectively. For in vivo assays, isoflurane (Isovet^®^, Isle Of Wight, UK) was obtained from B. Braun VetCare (Barcelona, Spain), oxytocin (Falcipart) from SYVA (León, Spain) and heparin (ROVI^®^, Madrid, Spain) from Laboratorios Farmacéuticos ROVI, S.A. (Madrid, Spain).

### 4.2. Cell Cultures

For in vitro assays, the polarized cell line Madin-Darby canine kidney (MDCK-II) and their murine Abcg2 and human ABCG2 transduced subclones, which were previously generated [63,64] and provided by A.H. Schinkel from the Netherlands Cancer Institute (Amsterdam, The Netherlands), were used.

Cell culture conditions have been previously described [65]. Briefly, cells were cultured in Dulbecco’s modified Eagle’s medium (DMEM) supplemented with GlutaMAX^TM^ (Life Technologies, Paisley, UK), penicillin (50 units/mL) and streptomycin (50 µg/mL) (Life Technologies, Grand Island, NY, USA) and 10% (*v*/*v*) fetal bovine serum (MP Biomedicals, Solon, OH, USA), at 37 °C in an atmosphere with 5% CO_2_. Cells were trypsinized every 3 to 4 days for subculturing, when a subconfluent state was reached.

### 4.3. Transcellular Transport Assays

Transcellular transport assays were performed as previously described [66], with minor modifications (Figure S1). Transduced MDCK-II cells were seeded as a monolayer on microporous membrane filters (3.0 μm pore size, 24 mm diameter; Transwell^®^ 3414; Costar, Corning, NY, USA) at a density of 1.0 x 10^6^ cells/well. Cells were grown for three days with daily medium replacement.

During the experiment, quality controls were performed in terms of monolayer tightness at the beginning and end of the assay using Millicell^®^ERS (Millipore, Burlington, MA, USA), and monolayer confluence was tested at the end of the experiment by means of Lucifer Yellow permeability [67]. Transport proficiency was simultaneously checked by testing a typical ABCG2 substrate (danofloxacin 10 μM) (Table S1).

Two hours before the start of the assay, medium at both the apical and basolateral side of the monolayer was replaced with 2 mL of prewarmed Opti-MEM medium (Life Technologies, Paisley, UK), either with or without the inhibitor Ko143 (1 µM) [66] (to verify that the potential transport was specifically due to ABCG2). The experiment began (t = 0 h) by replacing the medium of both compartments with fresh Opti-MEM medium with or without inhibitor and containing 10 µM lumichrome. Cells were incubated at 37 °C in an atmosphere with 5% CO_2_ and 100 µL aliquots were taken at 1, 2 and 3 h on the opposite side to where lumichrome had been added. This volume was replaced with fresh Opti-MEM medium. Finally, at t = 4 h 600 µL aliquots were taken from the apical and basal compartments of each well. All samples were stored at −20 °C until being analyzed by high-performance liquid chromatography (HPLC), as described below.

Lumichrome appearance in the opposite compartment is presented as the fraction of total compound added at the beginning of the experiment and expressed as percentage. The relative efflux transport ratio was calculated as the basal to apical directed transport percentage divided by the apical to basal directed transport percentage at 4 h.

### 4.4. Animals

Animals were handled according to institutional and ARRIVE guidelines complying with European legislation (2010/63/EU). Experimental procedures were approved by the Animal Care and Use Committee of the University of León and the Junta de Castilla y León (ULE_010_2021). Abcg2^-/-^ and wild-type mice were used, all of >99% FVB/N genetic background, generated and kindly supplied by A. H. Schinkel (The Netherlands Cancer Institute) [68].

All animals, which were aged from 9 to 14 weeks and weighed 30 g, were kept in a temperature-controlled environment with a cycle of 12 h of light and 12 h of darkness and received a standard diet and water ad libitum.

#### 4.4.1. Plasma and Tissue Distribution

For in vivo assays, 10 mg/kg lumichrome, 200 μL of compound solution [appropriate concentration in 6% (*v*/*v*) ethanol, 42% (*v*/*v*) polyethylene glycol 400 and 52% (*v*/*v*) saline solution] per 30 g of body weight, was intraperitoneally administrated.

Blood samples were collected at different time points (5, 10, 20, 30, 45, 60, 90 and 120 min) by cardiac puncture under anesthesia with isoflurane. Organs were harvested at the 60 min time point. All animals were euthanized by cervical dislocation at the end of the experimental procedure. Heparinized blood samples were centrifuged immediately at 3000× *g* for 15 min. Plasma and organs were stored at −20 °C until HPLC analysis. Three to ten animals were used for each time point.

#### 4.4.2. Milk Secretion Experiments

Pups of approximately 10 days old were separated from their mothers four hours before the onset of the experiment. Lumichrome 10 mg/kg solution, prepared as described above, was administered intraperitoneally, at 200 μL per 30 g of body weight. Milk secretion was stimulated by oxytocin (200 μL of a 1 UI/mL solution), subcutaneously injected to lactating mice 10 min before sample collection. Then, 30 min after lumichrome administration, blood and milk samples from the mammary gland were collected by retro-orbital plexus puncture and gentle pinching around the nipple using capillaries, respectively, under anesthesia with isoflurane. Animals were euthanized by cervical dislocation at the end of the experiment. Four to five animals were used for each group.

Heparinized blood samples were centrifuged immediately at 3000× *g* for 15 min to obtain plasma. Milk and plasma were also stored at −20 °C until HPLC analysis.

### 4.5. High-Performance Liquid Chromatographic Analysis

Samples were analyzed in a chromatographic system, which consisted of a Waters 2695 separation module and a Waters 2998 UV photodiode array detector.

Lumichrome was determined as previously described [37], with minor modifications. Tissue samples were homogenized with a mixture of 50% water:50% methanol with 0.5% HCl; 1 mL of solution per 0.1 g of organ was used. To each 100 µL of plasma, milk or tissue homogenate, 10 µL of an albendazole-2-aminosulfone solution (100 µg/mL) was added as an internal standard and 400 µL of acetonitrile was also added in order to precipitate proteins. After 10 min of being horizontally vortexed, samples were centrifuged at 10,500× *g* for 10 min at 4 °C. Supernatants were evaporated to dryness at 40 °C under a stream of nitrogen. Samples were reconstituted in 100 µL of cold methanol and injected into the HPLC system. However, samples for in vitro assays were directly injected into the HPLC system.

The chromatographic system used for sample analysis consisted of a Waters 2695 separation module and a Waters 2998 UV photodiode array detector. Separation of samples was performed on an analytical reversed-phase column (5 µm particle size, 250 × 4.6 mm, EVO C18 100 Å, Phenomenex^®^, Torrance, CA, USA). The mobile phase used for in vitro samples was trifluoroacetic acid 0.04%:methanol (40:60), whereas for in vivo samples, it was ammonium acetate 25 mM (pH 5):acetonitrile (85:15). In both cases, the flow rate was set to 1.00 mL/min and UV absorbance was measured at 260.3 nm.

Standard samples of lumichrome in the appropriate drug-free matrix were prepared yielding a concentration range of 0.0195–5 µg/mL for culture, plasma and tissue samples and 0.078–2.5 µg/mL for milk samples. Coefficients of correlation for culture and milk samples were above 0.99, whereas in plasma samples they ranged between 0.98 and 0.99, and in tissue samples between 0.94 and 0.99.

The limit of quantification (LOQ) and limit of detection (LOD) were calculated as described by Taverniers et al. [69]. LOQ was 0.008 µg/mL and LOD 0.003 µg/mL for cell culture samples; LOQ was 0.042 µg/mL and LOD 0.022 µg/mL for plasma samples; LOQ was 0.046 µg/mL and LOD 0.023 µg/mL for milk samples and for tissues, LOQ was 0.019–0.061 µg/mL and LOD 0.011–0.040 µg/mL.

### 4.6. Statistical Analysis

Statistical analysis was carried out using SPSS Statistics software (v. 26.0; IBM, Armonk, New York, NY, USA). The Shapiro–Wilk test was performed to check normal distribution. Comparison between groups was made applying the two-tailed unpaired Student’s t-test or the Mann–Whitney U test to normally or not-normally distributed data, respectively. *p* value ≤ 0.05 indicates statistically significant differences between groups.

## Figures and Tables

**Table 1:** Relative efflux transport ratio (apically directed translocation percentage divided by basolaterally directed translocation percentage) at 4 h for lumichrome (10 µM) in parental MDCK-II cells and their subclones transduced with murine (mAbcg2) or human ABCG2 (hABCG2) variant, either in the absence (−Ko143) or the presence of the ABCG2-specific inhibitor Ko143 (1 µM) (*n* ≥ 3).

	−Ko143	+Ko143
MDCK-II	0.85 ± 0.13	0.95 ± 0.03
MDCK-II mAbcg2	13.81 ± 3.25 *	1.03 ± 0.04
MDCK-II hABCG2	2.57 ± 0.25 *	1.08 ± 0.08

Results are shown as mean ± S.D. (*) *p* ≤ 0.05: significant differences in transport ratio compared with parental MDCK-II cells.

## Data Availability

The data supporting the reported results are available from the corresponding author upon request.

## Supplementary Materials

The following supporting information can be downloaded at: https://www.mdpi.com/article/10.3390/ijms25189884/s1.
