# Supplementary material for: The ABCG2 Transporter Affects Plasma Levels, Tissue Distribution and Milk Secretion of Lumichrome, a Natural Derivative of Riboflavin
Source: Int J Mol Sci. 2024 Sep 13;25(18):9884. doi: 10.3390/ijms25189884 (PMC11431963; doi:10.3390/ijms25189884)
Supplement: Supplementary file 1 [file ijms-25-09884-s001.zip › ijms-3164772-supplementary.pdf]

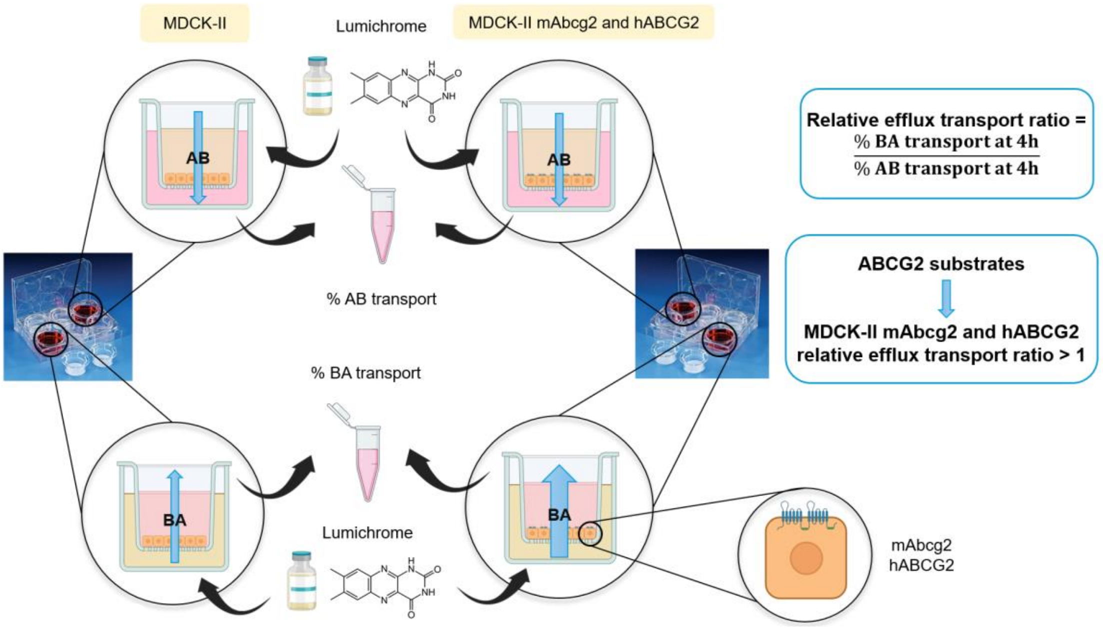

Supplementary Figure S1. Transcellular transport assays. ABCG2 overexpressed in the apical membrane of the polarized cells transports its substrates toward the apical direction. In this way, vectorial transport in ABCG2 transduced cells is greater in the basolateral-to-apical (BA) direction than in the apical-to-basolateral direction (AB). Transport from the basal to the apical compartment (BA) was evaluated by adding lumichrome in the basal compartment and aliquots were taken from the apical compartment. Transport from the apical to the basal compartment (AB) was tested by adding lumichrome in the apical compartment and aliquots were taken from the basal compartment. Created in BioRender.com.

Supplementary Table S1. Basolaterally directed translocation (BA transport) and apically directed translocation (AB transport), both expressed as a percentage, and relative efflux transport ratio at 4 h for danofloxacin (positive control) in parental MDCK-II cells and their subclones transduced with murine (mAbcg2) and human (hABCG2) variant of ABCG2.

	**BA transport (%)**	**AB transport (%)**	**Transport ratio (BA/AB)**
MDCK-II	21.82 ± 1.79	22.84 ± 2.99	0.96 ± 0.08
MDCK-II mABCG2	45.77 ± 6.49	5.30 ± 1.97	9.24 ± 2.59*
MDCK-II hABCG2	33.94 ± 4.07	17.22 ± 2.98	2.04 ± 0.57*

Results are shown as mean ± S.D.

* $p \leq 0.05$: significant differences in transport ratio compared to parental MDCK-II cells.
